# Confronting hidden COVID-19 burden: a telemedical solution for elective urological outpatient clinics

**DOI:** 10.1007/s15010-020-01511-7

**Published:** 2020-09-06

**Authors:** Thilo Westhofen, Giuseppe Magistro, Simon Lennartz, Jozefina Casuscelli, Christian Stief, Severin Rodler

**Affiliations:** 1 Department of Urology, Ludwig-Maximilians-University of Munich, Marchioninistrasse 15, 81377 Munich, Germany; 2 Faculty of Medicine and University Hospital Cologne, Institute for Diagnostic and Interventional Radiology, University of Cologne, Cologne, Germany; 3 Department of Radiology, Massachusetts General Hospital, Harvard Medical School, 55 Fruit St, White 270, Boston, MA 02114 USA

**Keywords:** COVID-19, Telehealth, Patient management, Elective patients

## Abstract

Maintaining high-quality care for urological patients is a challenge during and after the Coronavirus disease 2019 (COVID-19) pandemic. We observe an increasing volume of postponed elective visits at our tertiary care hospital, holding the risk for deterioration of non-emergency disease conditions. As it is unclear for how long the pandemic will last, we propose to implement telehealth as a solution to provide regular symptom monitoring compatible with social distancing guidelines during the pandemic and beyond. Telemedical assessment and prioritizing of high-risk patients for individual consults at outpatient services will have to be aligned with available outpatient capacity and local outbreak severity.

**Electronic supplementary material:**

The online version of this article (10.1007/s15010-020-01511-7) contains supplementary material, which is available to authorized users.

## Background

Initially discovered in Wuhan, the *Severe Acute Respiratory Syndrome Corona Virus 2* (SARS-CoV-2) [1] has rapidly spread around the world and hence has been declared a pandemic by the world health organization (WHO) on March 11, 2020 [2]. As the outbreak forced health care systems to reallocate medical resources to provide capacities for the overwhelming surge of COVID-19 patients, the majority of elective urological outpatient procedures at our hospital have been suspended and postponed.

With more than 155,000 confirmed cases and more than 6000 reported deaths in Germany as of April 29, 2020 [3] the current efforts are mostly directed towards managing the acute situation and developing curative treatments [4] or vaccinations [5]. Yet, adapting and maintaining outpatient care is another arising challenge [6]. As the majority of urological private practices shut down during the first COVID-19 surge, the burden of postponed elective treatments on our healthcare systems dramatically grows. Moreover, as prolonged or intermittent social distancing might be necessary until as late as 2022 [7], solutions for outpatient care should be suitable for long-term implementation, if required.

As a large tertiary referral center, we sought to establish a strategy to overcome the anticipated increase in patient volume and provide adapted outpatient care in the post-pandemic phase.

### Methods

Anonymized patient data were obtained from digital patient records of all elective patients appointed for urology outpatient visits between 16th March and 12th April. A basic COVID-19 risk assesses for all patients was performed in terms of travel history, fever, respiratory symptoms, suspected or confirmed COVID-19 status, and primary health care referrals. In accordance with institutional guidelines, the institutional review board (Ethikkomission der Ludwig-Maximilian-Universität München) has reviewed the project design and waived need for approval (Reference number: 20-340). Descriptive statistics were performed, Fisher’s exact test and Mann–Whitney *U* test were applied for univariate analyses of categorical variables and continuous variables, respectively.

### Patient characteristics

**Fig. 1:**
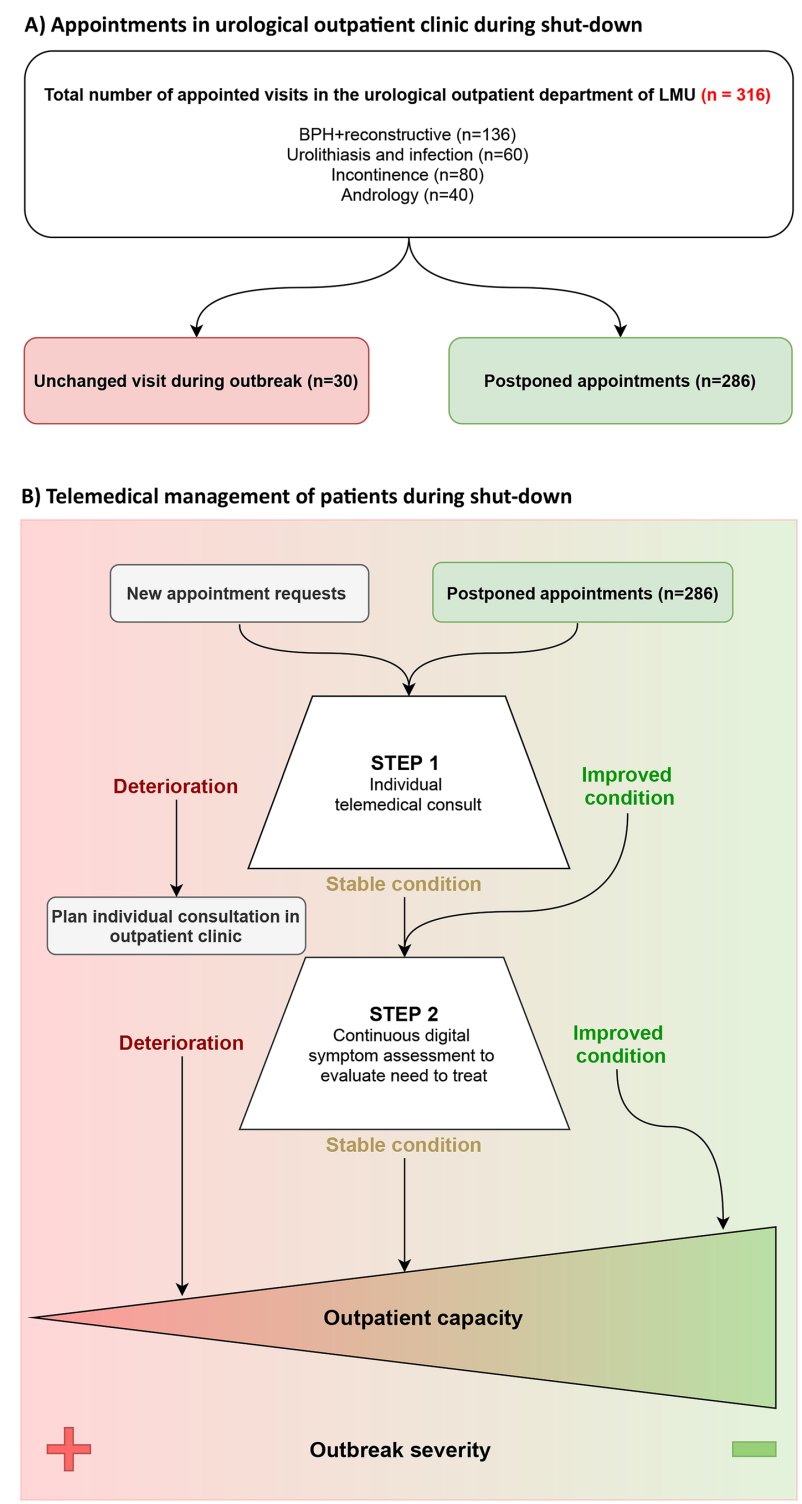
**a** Appointments in the urological outpatient clinic during shutdown. **b** Telemedical management of patients during shutdown

Patient characteristics are summarized in Table 1. 316 patients with non-malignant urological conditions, mainly by referral due to complexity or chronic reoccurrence of disease were scheduled for an appointment at our specialized urological outpatient clinic for the study period of 4 weeks. 30 patients (9.49%) were seen as scheduled whereas 286 (90.51%) were rescheduled (Fig. 1a). Median age of all patients was 64 years (IQR 51–75). 54.7% of the patients were classified as ASA ≤ 2, 45.3% were ASA > 2. The overall rate of patients with comorbidities was significantly higher in BPH (70.58% (96/136)) than in urolithiasis (56.67% (34/60)) or incontinence (48.75% (39/80)). In Andrology rate of comorbid patients was the lowest (18.33% (11/60)) (each *p* < 0.05, respectively) (Table 1).

**Table 1:** Patient characteristics of elective patients with appointed visits in the urological outpatient clinic

	BPH + reconstructive (*n* = 136)		Urolithiasis + infection (*n* = 60)		Incontinence (*n* = 80)		Andrology (*n* = 40)	
Age								
Median	63		62		72		41	
Range	52–71		52–75		68–78		33–53	
	*n*	%	*n*	%	*n*	%	*n*	%
Sex								
Male	129	94.8	33	55.6	68	85.2	36	90.0
Female	7	5.2	27	44.4	12	14.8	4	10.0
ASA status								
≤ 2	71	52.2	36	60.0	33	41.7	36	88.9
> 2	65	47.8	24	40.0	47	58.3	4	11.1
Chronic underlying condition								
Hypertension	40	29.2	4	7.1	10	12.1	0	0.0
Cardiac disease	40	29.2	17	28.6	22	27.3	0	0.0
Pulmonary disease	28	20.8	0	0.0	5	6.1	3	7.7
Renal disease	11	8.3	13	21.4	5	6.1	3	7.7
Oncological disease	11	8.3	21	35.7	5	6.1	0	0.0
Obesity	23	16.7	0	0.0	12	15.2	0	0.0
Diabetes	11	8.3	4	7.1	12	15.2	0	0.0
Compromized immun system	23	16.7	13	21.4	10	12.1	0	0.0
Continuation of treatment								
Unchanged	10	7.4	7	11.7	9	11.3	4	10.0
Appointment postponed	126	92.6	53	88.3	71	88.8	36	90.0

## Postponement of elective patients: recent experience and future challenges

### Adapted procedures in outpatient clinics of a university hospital

As a reaction to COVID-19 outbreak in Munich, the Ludwig-Maximilian-University Hospital implemented guidelines to manage the upcoming challenges. In accordance with regulations by health care officials, immediate cessation of deferrable consultations was arranged. Appointments were rescheduled and only in cases of immediate urgency, such as urinary retention or severe infections, consultation in our outpatient clinic was authorized (Fig. 1a).

### Unmet amount of elective patients

As COVID-19 regulations jeopardized treatment of elective patients in our urological outpatient clinic, we faced the postponement of 286 out of 316 elective patients (Table 1)within 4 weeks between 16th March and 12th April. Only 30 patients were seen as scheduled. Scaled up by constantly incoming new appointment requests, the burden of deferred elective patients critically rises with ongoing of the COVID-19 pandemic—particularly, as its end and the point of returning to pre-pandemic routines are uncertain [7]. In a tertiary care setting, we expect deferred elective patients to reach critical burden even faster than for higher prioritized oncologic patients [8], such that catching up on postponed visits may exceed capacities many times over. As treatment capacities cannot be expanded beyond a certain degree and distribution of health resources will likely become competitive, we will most likely face a supply shortfall for elective patients. As secondary care is mostly shut down, transfer to a resident urologist, as previously described for uro-oncology patients [6], is not an option.

### COVID-19 side effects: elective turning into urgent

Without adequate treatment, even chronic or non-emergency disease conditions can deteriorate and turn into acute problems. With the postponement of treatment, lower urinary tract symptoms (LUTS) due to benign prostate hyperplasia (BPH) decompensate, leading to acute urinary retention with an enhanced risk for renal failure and urosepsis. Likewise, a known urolithiasis, prone to infections or chronic urinary tract infections (UTI) can deteriorate with increased risk for severe infections and urosepsis. In tertiary care centers, the deferment of treatment may cause severe physical impairment, as many patients have reduced physiological resilience due to comorbidities and high complexity of cases. Conversely, in andrology, the urgency may arise from a certain window of opportunity for fertility assessment and treatment.

## Preventing the overload: continuous reassessment and early reopening of outpatient services

With the burden of postponed elective patients increasing, we see the need for a risk and urgency adapted continuous reassessment of patients during the pandemic. To sustain high standards of medical care and simultaneously ensure social distancing, patients should be followed up remotely during the COVID-19 pandemic and beyond, if required. The best solution in our opinion is telehealth measures as regular symptom assessment to check for deterioration of the electively managed disease condition. We propose implementation of a simple telemedical two-step algorithm. In STEP 1, which substitutes scheduled appointments, patients should be consulted by trained physicians over the phone to assess the symptoms and the level of suffering, applying standardized scores. Only in case of acute deterioration, consultation in outpatient clinics should be authorized. For STEP 2, patient apps for outcome recording should be implemented to provide continuous symptom control, as they have proven efficacy in oncology where they have already been established [9]. This steady reassessment allows the necessary triage of elective patients. In doing so, the forthcoming caseload of elective patients could be controlled and mitigated, preventing outpatient centers to be overwhelmed. As major hurdles, reimbursement and regulatory issues are perceived, but governments might be willing to adapt during this pandemic [10]. Assisted by telemedical symptom assessment, triage for appointments should be based on the probability of deterioration, the current outpatient capacity and local risk for SARS-CoV-2 infections (Fig. 1b). Furthermore, we must strive for the early reopening of outpatient clinics as soon as the curve flattens to avoid later short cuts and deterioration in the medical service. As patients should be already virtually reassessed for potential progression of their diseases during the pandemic, a restart should be performed following the same triaging principles. Probability of deterioration, time aspect in andrology and outpatient capacity should be used to prioritize patients.

### Experience after re-opening of outpatient clinic

The current burden of postponed elective treatments 8 weeks after gradually reopening our outpatient clinic is shown in supplemental Fig. 1. A total 2.5% (8/316) of the patients were admitted due to emergencies, 41.5% (131/316) of the patients were rescheduled and appointed and 56.0% (177/316) of the postponed patients remain to be appointed (Suppl. Figure 1).

## Conclusion

Providing a high standard of medical care for elective outpatients is a challenge as the COVID-19 pandemic prolongs. The implementation of strategies to maintain surveillance and care for patients with deferrable requests appears essential at this time to prevent undersupply in the post-COVID-19 era. Telehealth should play a crucial role to ensure safe and efficient reevaluation of patients. Partial reopening of outpatient clinics should be pursued as early as possible, taking into account patient risk and urgency of individual requests.

## Electronic supplementary material

Below is the link to the electronic supplementary material.

Supplementary file1Supplemental Figure 1: Experience 8 weeks after gradually re-opening of urological outpatient clinic (TIF 595 kb)

## Data Availability

Data are available for bona fide researchers who request it from the authors.
