# Supplementary figures and images for: Confronting hidden COVID-19 burden: a telemedical solution for elective urological outpatient clinics
Source: Infection. 2020 Sep 6;48(6):935–9. doi: 10.1007/s15010-020-01511-7 (PMC7474800; doi:10.1007/s15010-020-01511-7)

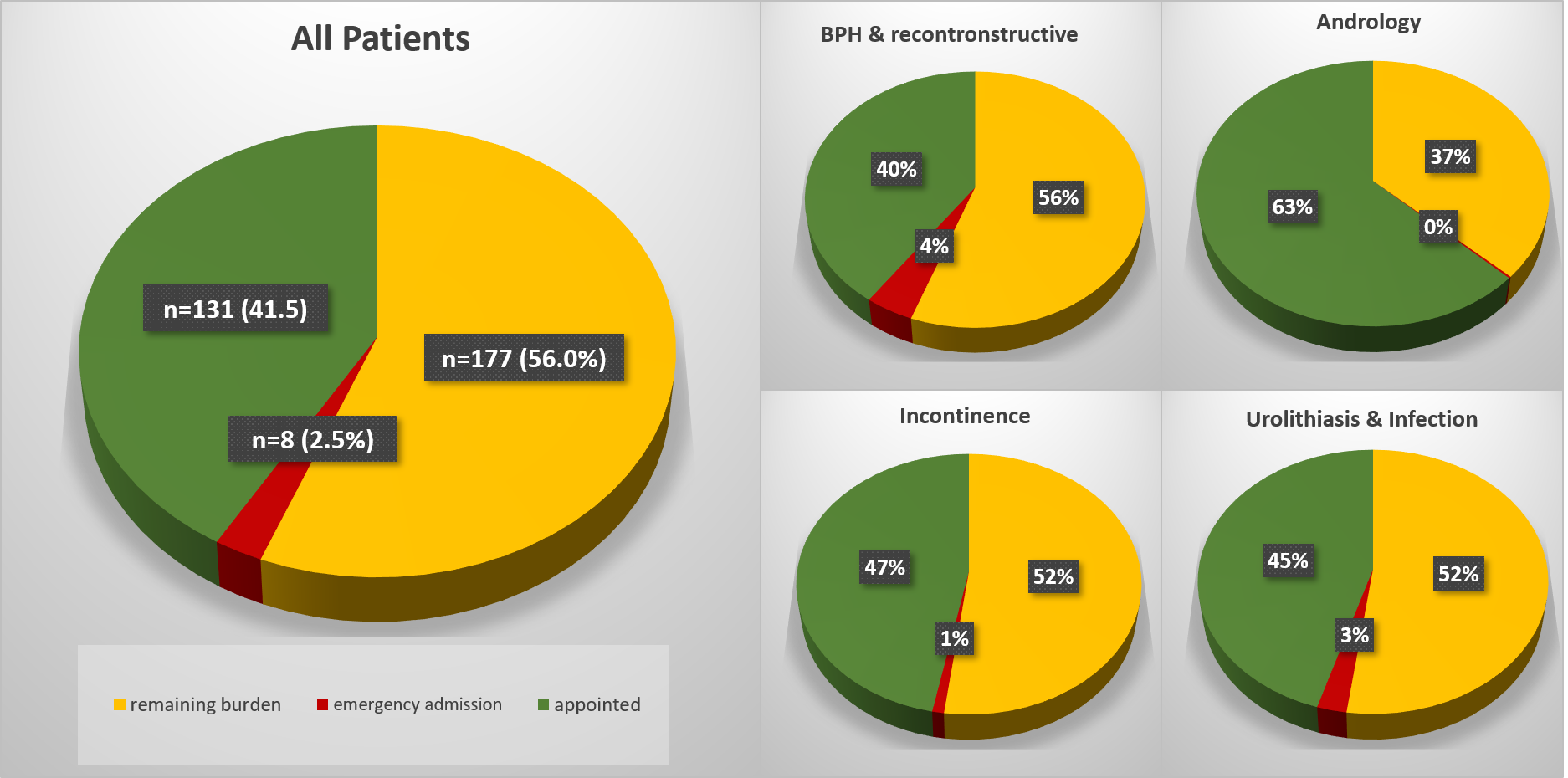

Supplement: Supplementary file 1 — Supplementary file1Supplemental Figure 1: Experience 8 weeks after gradually re-opening of urological outpatient clinic (TIF 595 kb) [file 15010_2020_1511_MOESM1_ESM.tif]
